# Supplementary material for: Social Media Use and Adolescents’ Sleep: A Longitudinal Study on the Protective Role of Parental Rules Regarding Internet Use before Sleep
Source: Int J Environ Res Public Health. 2021 Feb 2;18(3):1346. doi: 10.3390/ijerph18031346 (PMC7907989; doi:10.3390/ijerph18031346)
Supplement: Supplementary file 1 [file ijerph-18-01346-s001.pdf]

Supplementary material.

Table S1: Correlations between all Study Variables.

Variable	1	2	3	4	5	6	7	8	9	10	11
1. Gender (ref = girls)	1.00										
2. Age	0.06**	1.00									
3. Educational level (ref = higher)	−0.17***	0.03	1.00								
4. Frequency social media use T1	−0.14***	0.12***	0.07***	1.00							
5. Problematic social media use T1	−0.06**	−0.05*	0.15***	0.31***	1.00						
6. Bedtime T1	0.11***	0.42***	0.09***	0.30***	0.17***	1.00					
7. Bedtime T2	0.15***	0.36***	0.04	0.29***	0.14***	0.60***	1.00				
8. Quality of sleep T1	0.09***	−0.04	0.03	−0.15***	−0.27***	−0.25***	−0.14***	1.00			
9. Quality of sleep T2	0.10***	−0.09***	0.02	−.14***	−0.17***	−0.22***	−0.27***	0.57***	1.00		
10. Parental rules Internet T1 (ref = no strict rules)	0.03	−0.20***	−0.01	−0.23***	−0.13***	−0.29***	−0.21***	0.14***	0.12***	1.00	
11. Parental rules smartphone T1 (ref = no strict rules)	0.04	−0.20***	0.06**	−0.17***	−0.10***	−0.33***	−0.26***	0.12***	0.10***	0.39***	1.00

Note. Spearman's Rho was used for correlations with gender, educational level, problematic social media use, parental rules regarding using the Internet in the hour before going to sleep and parental rules regarding taking the smartphone to the bedroom when going to sleep. Pearson Correlation was used for correlations with all other variables. * $p < .05$. ** $p < .01$. ***$p \leq .001$.
